# Flipped Classroom in Restorative Dentistry: A First Test Influenced by the Covid-19 Pandemic

**DOI:** 10.3290/j.ohpd.b3276187

**Published:** 2022-08-03

**Authors:** Canan Özcan

**Affiliations:** a Assistant Lecturer, Dentist, Department of Conservative Dentistry, School of Odontology, University of Reims Champagne-Ardenne, Reims, France. Conceptualisation, methodology, validation, formal analysis, data curation, investigation, wrote, reviewed and edited manuscript.

**Keywords:** Covid-19, flipped classroom, lower cognitive level, restorative dentistry

## Abstract

**Purpose:**

This study compared the success of dental students with flipped classroom and traditional classroom learning in the restorative dentistry course for the first time at the Faculty of Dentistry of Reims during the 2019-2020 academic year, influenced by the Covid-19 pandemic, and analysed the correlation with students’ feelings. The use of an active learning method can improve success during final exams and increase the motivation of students.

**Materials and Methods:**

The same teacher taught half of the restorative dentistry course in the flipped classroom approach and the other half as traditional classroom. For the flipped classroom, students were required to complete their homework online before the face-to-face sessions. An exam at the beginning and the end of the semester was conducted with questions about concepts learned with each learning method. Statistical analysis was performed using a t-test at the 0.05 significance level. A questionnaire on satisfaction was conducted to determine the students’ opinion on this new learning method in the flipped classroom compared to the traditional classroom.

**Results:**

The flipped classroom learning method enabled students to achieve better results on the final exam, with a statistically significant difference compared to traditional classroom learning. Student responses to the satisfaction questionnaire showed an increase in motivation and interest in the lessons and correspond to the increase in exam success.

**Conclusion:**

The use of the flipped classroom for lower cognitive-level activities is more appreciated by students and yields better results than knowledge acquisition in the traditional classroom.

In dentistry, attempts to introduce new methods of student-focused teaching are increasingly frequent, especially in graduate and postgraduate studies. In the first cycle, traditional courses are still the most common. These are simpler, less expensive to set up, and also make it possible to work with many students at the same time in a large auditorium. These teaching methods relegate a very passive role to students who find it difficult to stay focused throughout the lesson without active involvement. In the fields of medicine and dentistry, the student – who is a future practitioner – must acquire skills in critical analysis, decision-making, synthesis, and practical skills concerning the acts to be performed with their patients. The literature shows that working with role-playing^37^ and group activities^2^ can improve results for these students. These active learning methods require time and teamwork on the part of teachers as well as some teaching experience. In terms of fundamentals, flipped classroom methods are more suitable for imparting knowledge.^4,13,17,26^ Students are placed at the centre of their own learning; they seek information instead of being spectators of a lesson given by the teacher. The transfer of knowledge begins before the course, which then becomes only a place of exchange to complete the missing or poorly understood information.^28^ There are different opinions about the flipped classroom, because the positive perception of students does not always lead to better outcomes^16,32^ compared with the traditional classroom; however, the flipped classroom allows students to gain motivation and better follow the lessons.^32^

The 2019-2020 academic year was marked by the Covid-19 pandemic and forced many universities to review their educational system to continue the transfer of knowledge.^31^ It has also led to the use of digital methods for distance learning for the first time in many universities. Courses had to be designed with active paedagogy to make the content interactive and more motivating to keep the students’ attention until the end of the course.

Flipped classroom teaching was implemented for the first time in the restorative dentistry course for second preclinical-year dental students at our faculty. The aim of this study was to observe the success of these students with flipped classroom teaching compared to traditional teaching and to measure the impact felt by the students during the closing of the University due to Covid-19.

## Materials and Methods

The local Institutional Review Board deemed the study exempt from review, but informed consent was obtained from all individuals included in this study.

The restorative dentistry course, taught in the second semester for second preclinical-year dental students at our school of dentistry in France, was modified to use a flipped classroom method.

**Fig 1:**
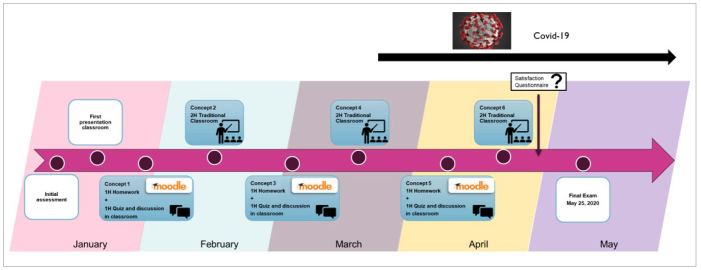
Presentation of the semester progression with the flipped classroom and traditional classroom for restorative dentistry courses.

The course on fundamentals of dental composites, initially delivered in a traditional auditorium classroom from January to May, was completely restructured to include a flipped classroom part and a traditional classroom part. The concepts covered in this course consisted of 6 parts: 1. learning the SI/STA (site and stage) classification for dental caries;^18^ 2. the various composites and the criteria of choice according to their compositions; 3. the criteria of choice of the shade of the composite; 4. the choice and the use of the composite curing light; 5. the criteria for choice of an adhesive according to their compositions and implementation steps; 6. the indications and contraindications for the placement of a composite restoration. When these courses were conducted in the traditional classroom, they required about 2 hours of lecture-hall time for each part, that is, a total of 12 hours of class time during the semester. For this study, the 6 parts were classified to form two groups of courses of equivalent difficulty and amount of knowledge to be imparted. Each group of lessons consisted of 3 parts and had to take a similar amount of time. The first group of courses was held in the flipped classroom approach and the second in the traditional classroom (Fig 1).

At the beginning of the semester, before the course was introduced, the students’ knowledge of these 6 courses was examined by means of 20 questions. Single best answer questions were used to simplify the exam. Ten questions dealt with the concepts that had to be learned with the flipped classroom and ten with the concepts of the traditional classroom part. These were concepts that the students were learning for the first time. We were therefore prepared to have a very low pass rate for this exam.

For the traditional class, nothing has been changed compared to previous years. Classes were held in an auditorium with the teacher in charge. The students learned about the concepts for 2 hours per class. No work upstream of this session was required of them. The PDF of the lesson was available online after each lesson and could be used as study material before the exam. Students could ask questions during the lesson or by e-mail after the lesson.

In the case of the flipped classroom, students had to complete an assignment before the class period. For this, a first face-to-face session took place at the beginning of the semester to explain to the students the principles of the flipped classroom and the tools that would be used. Then, for each concept taught by this learning method, the students had to connect to the learning management system ‘Moodle’ (modular object-oriented dynamic learning environment) proposed by the university and take note of the different paedagogical activities put online. These activities were:

- reading downloadable documents accessible via links:
- watching short videos on the concepts to be learned and answers questions about the;
- use of interactive learning capsules made with the HTML5 Plugin H5P (https://h5p.org/) with flash cards, hotspot images, true/false quizzes or synthesis tools, etc. This plug-in is an open-source tool integrated directly in Moodle (Perth, Western Australia, Australia) and used to create an interactive learning object.^33^

This distance-learning time before in-class time was estimated at 1 hour for each part. Throughout this distance learning, students had the opportunity to ask the teacher questions directly by e-mail as for the traditional class. This homework was supplemented by 1 hour of classroom work starting with a formative assessment through an online quiz (Wooclap; Brussels, Belgium).

The teacher posted the questions on the board, the students had to connect to the quiz with their smartphone or laptop using the link given by the teacher. They could then answer as the quiz progressed. A countdown timer allowed them to answer within a certain time. The teacher could see the percentage of selected answers live. Following the quiz, a correction for each question was made, and students could ask questions and interact with the teacher.

Unfortunately, the second semester of the 2019-2020 academic year was cut short by the onset of the Covid-19 pandemic. In France, from 16 March 2020, a national lockdown was declared, similar to other European countries, which also applied to dental institute teaching. Universities were closed and courses had to be delivered by distance learning. This meant that we had to transform classroom courses into video conferences. In our case, only one traditional class and one flipped class period were changed to a video-conference session.^12,2576^ For the rest of the teaching, nothing changed because knowledge was already acquired through interactive online activities.^31^

A satisfaction questionnaire was put online during the month of April 2020 to collect students’ opinions on these two types of learning.

At the end of the semester, an exam with 20 questions was used to assess the students’ performance. The questions in this exam were similar to those at the beginning of the semester, but with an additional degree of difficulty. Most of the questions were multiple choice or short answer questions. There were 10 questions on concepts learned in the flipped classroom and 10 questions on concepts learned in the traditional classroom.

Of the 86 students who took the exam, only 51 were included in the study. Repeaters who had already learned these concepts the previous year, as well as students who were absent from all classroom sessions. were excluded from the study. Only those students who were able to take advantage of all the online and in-class learning materials were included.

The statistical analysis of the results was carried out with a t-test using R software (version 4.0.3) with the significate level set at 0.05.

## Results

**Fig 2:**
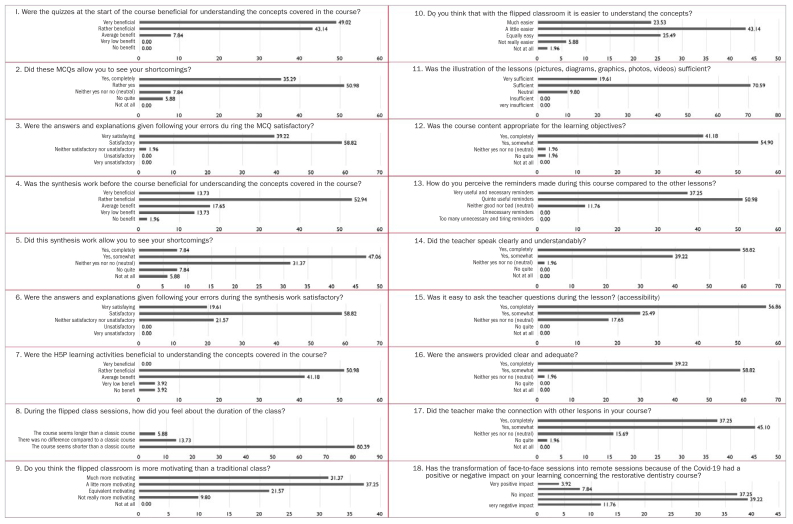
Graphical representation of the students, answers to the satisfaction questionnaire (in %).

Figure 2 shows the satisfaction questionnaire answers. Overall, students felt that the MCQs, quizzes, synthesis tools, and H5P learning capsules helped them to better understand the concepts and fill in gaps. They expressed more motivation and less frustration when learning compared to traditional learning. They felt that the answers provided by teachers were relevant to their questions and met their expectations. However, they felt that the transformation of face-to-face courses into video conferences during the pandemic had a negative impact on their learning. When questioned orally on the reasons for this negative impact after obtaining our results, the students affirmed that the online courses could sometimes extend over the whole day. The students spent their day in front of their computer screen without any real interaction. The strict 3-month confinement period therefore ended up reducing their motivation to learn because they were more tempted to do something else on the internet than listen to the courses. This is consistent with the findings of Meeter et al,^22^ who indicate that lack of social interaction was the cause most often cited as a reason for loss of motivation.

**Fig 3:**
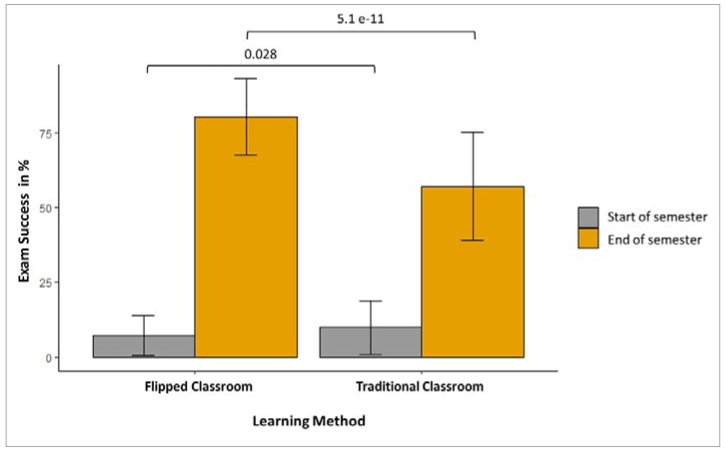
Results (in %) of the initial assessment and final exam with the flipped classroom and the traditional classroom learning methods.

Figure 3 shows the success rate of students in the initial and final exams according to the learning method used. The pass rate with the flipped classroom method was 7.2% for the initial assessment and 80.5% for the final exam, while with the traditional classroom it was 9.8% for the initial assessment and 57% for the final exam. The students therefore had a statistically significantly higher success rate on questions concerning the concepts covered in the flipped classroom.

## Discussion

Thanks to the extensive media coverage of flipped classroom teaching in many areas, more and more teachers are trying to switch to new methods of learning and teaching. In medicine, the literature is overflowing with positive and encouraging flipped classrooms examples, reporting that most students prefer this method to the traditional classroom.^8,9,20,35^

The aim of our study was to observe the effects of flipped classroom learning on the success rate of students dealing with concepts they were learning for the first time. This test was also a first for restorative dentistry courses in our faculty, where this type of teaching is still in its infancy.

The flipped classroom concept introduces another dimension to teaching. The student becomes an active partner in his or her own knowledge development. Gone are the days of passive learning where students only had to be present in the classroom and wait for things to happen. The traditional teacher-centred classroom inevitably bores students and deprives them of developing essential skills such as critical thinking, problem solving and communication.^11,14,23,27^ The potential of the flipped classroom has been recognised in the literature.^1,5,8,9,20,35,38^

Initially, examples of alternatives consisting of reorganising teaching in and out of the classroom emerged,^15,21,26,34^ but they have been questioned by many university teachers and practitioners. In all learning, the improvement offered by flipped classroom learning lies in the addition of tasks to be performed by the teachers and students during face-to-face sessions in class or during work at home.^5,7^ It cannot just be the provision of courses to be read on a digital medium.

In our study, the use of the flipped classroom and the traditional classroom for the same class of students in the same course allowed us to exclude cohort or conceptual learning bias. Students were able to give feedback without being disturbed by the change of teacher or the change of discipline.

In the final exam, students gave more correct answers to questions about concepts learned with the flipped classroom method. The examination at the beginning of the semester was intended to show that the students did not have any prior knowledge that could have biased them in this study.

The opinions given to the different questions in the satisfaction questionnaire also showed that the students were encouraged by the flipped classroom learning. Their perception of the duration of the course showed that they were less bored during this session. They thought that the course seemed shorter with a flipped classroom compared to the traditional classroom. Their impressions were positive about comprehension, which they found easier. They also felt that they gained a real benefit from the different activities offered. It was this combination that enabled them to follow the lessons better in the flipped classroom and to achieve the objectives of each lesson.

The answers given to the satisfaction questionnaire showed the importance of adequate answers provided by the teacher to questions students may have. This is a set of elements that allows understanding of the concepts taught. The answers provided, as well as reminders about concepts to be learned, completed the content of the course. It was obvious that it was not a question of making a presentation only with text that would be difficult to read in its entirety and that would bore the students. In the flipped classroom we offered, the course content was usually composed of illustrations. The students also appreciated this type of presentation and found it helpful in achieving the learning objectives.

The results obtained in the final exam were objective proof of the success and truthfulness of the feelings reported by the students. They achieved better results with the flipped classroom learning method. This shows that this more motivating method generates a real learning benefit. Prober et al^30^ also made the same conclusion in a study with first-year biochemistry students. They observed an increase in student participation ranging from 30% to 80% when the course was taught as a flipped classroom. Lew’s study about student’s individual evaluation after a flipped classroom application in emergency medicine showed an increase of participation and knowledge.^19^ In our study and in the literature, the role of the teacher and the interaction with the teacher was very important to the students. Whelan et al^39^ reported that students felt a lack of direction and support when the teacher in the practical anatomy class dominated the discussion and did not apply an active teaching method. Our students indicated by their responses that the adequacy of teachers’ answers help them to progress by filling their learning gaps. With active teaching methods, the teacher must abandon her/his central role and accept that the attention is focused on the students.^36^ S/he should gradually transfer responsibility for learning to students.^36^ This is an absolute prerequisite for the success of these teaching methods.^35^ Obviously, this is not easy, as the faculty themselves have received and applied traditional teaching for years. In active teaching, the teacher becomes a collaborator with student learning.^34^ They are on a peer-to-peer level that is sometimes difficult to accept for teachers used to having a monopoly on discussion in class.^3^ It is also a more energy-intensive position for students, because they must participate in activities.^26,35^ Unlike traditional teaching, which relied solely on the responsibility of the teacher, the student is involved in all phases of his or her learning and is thus empowered.

Morton and Colbert-Getz^24^ showed that the use of an active method of learning, with lower cognitive levels of Bloom’s taxonomy, e.g. recall of knowledge, had no impact on students’ examination results. But when these methods were used for higher cognitive levels related to analysis and synthesis of this knowledge, performance was better. However, our study shows that the flipped classroom can also be a source of success for novice students who are in the knowledge acquisition phase. The use of this new method in comparison with the traditional classroom in the same course with the same teacher eradicates bias and allows the effect of the learning method to be directly visualised. Finally, the correlation between the students’ feelings and the results obtained shows that success follows motivation to learn.

Today, the implementation of a flipped classroom is accompanied by computer use and the internet. This makes it possible to keep in touch with the students despite the distance and, above all, to have regular monitoring of the work done. The educational platforms used in universities allow the implementation of multiple educational activities to be carried out in class or at home. In the classroom, this increases interactivity, and at home, it is a self-assessment tool for students. However, in spite of all these tools, we notice that students still need visual contact with the teacher. In our study, this was demonstrated by the question about the changes related to Covid-19. All classroom activities were already being carried out using interactive online methods from the beginning. When the universities were closed due to the pandemic, the only change was the transformation of face-to-face classes to video conferencing. This change was perceived as negative by the students. They felt that not being face-to-face with the teacher during the course had a negative impact on the learning process. Putting a face to the speaker who can also see the reactions of the audience was interpreted as necessary for better learning. This face-to-face interaction was the second pillar described by Doolittle for collaborative learning.^10^ It is also one of the two components of Prince’s description of active learning.^29^ Teachers also need to acquire the skills to make optimal use of distance learning and communication tools to better detect students’ needs and provide rapid responses and support. The sudden onset of the pandemic did not allow time for this learning to take place, but all educational institutions did their best and shared the same conviction that learning should not stop.^6^

## Conclusion

The first implementation of the flipped classroom in restorative dentistry courses in the 2019/2020 academic year was a success, despite the disruption of the Covid-19 pandemic. Student feedback on this new teaching method showed that the interactivity of a course enabled better learning by increasing student interest in their courses. These results also showed that the teacher cannot be totally replaced, and that the teacher’s role is crucial in any teaching method.
